# Real-world analysis on the use of gamma-hydroxybutyric acid for alcohol withdrawal syndrome in hospitalized patients with diagnosis of cirrhosis

**DOI:** 10.1007/s11739-024-03761-x

**Published:** 2024-09-09

**Authors:** Monica Salomoni, Andrea Missanelli, Giada Crescioli, Cecilia Lanzi, Arianna Totti, Lorenzo Losso, Stefano Gitto, Roberto Bonaiuti, Alfredo Vannacci, Niccolò Lombardi, Guido Mannaioni

**Affiliations:** 1 Toxicology Unit and Poison Control Center, Careggi University Hospital, Florence, Italy; 2 Department of Neurosciences, Psychology, Drug Research and Child Health, Section of Pharmacology and Toxicology, University of Florence, Viale G. Pieraccini, 6, 50139 Florence, Italy; 3 Tuscan Regional Centre of Pharmacovigilance, Florence, Italy; 4 Department of Experimental and Clinical Medicine, University of Florence, Florence, Italy

**Keywords:** Alcohol withdrawal syndrome, Clinical practice, Gamma-hydroxybutyric acid, Liver cirrhosis, Sodium oxybate, Toxicology

## Abstract

The present real-world analysis aimed to evaluate and describe the use of gamma-hydroxybutyric acid (GHB) for alcohol withdrawal syndrome (AWS) in hospitalized patients with diagnosis of liver cirrhosis. An 11-year observational retrospective study on patients affected by liver cirrhosis and alcohol use disorder (AUD) was performed using data from the Medical Toxicology Unit of Careggi University Hospital in Florence (Italy). A multivariate logistic regression was performed to estimate the probability of having a CIWA-Ar _Max_ 3–4 during hospitalization, an AWS length  > 36 h, a hospitalization > 9 days, and the probability of developing drowsiness. A total of 166 AUD patients were included, of these 77 received GHB (70.13% within the first day of hospitalization) and 89 were treated without GHB. The majority were  ≥ 40 years of age (87.35%) and males (80.12%). GHB patients were more likely to have a CIWA-Ar _Max_ 3–4 during hospitalization (OR 3.76 [CI 95% 1.02–13.85]), and a longer hospitalization (OR 3.08 [95% CI 1.23–7.71]). Early GHB administration decreased the probability of CIWA-Ar _Max_ worsening (OR 0.06 [95% CI 0.01–0.49]). GHB dose  ≥ 100 mg/kg was not associated with the occurrence of drowsiness. Patients exposed to other sedative agents were more likely to experience drowsiness (OR 7.22 [95% CI 1.46–35.61]). The present real-world analysis underlines that GHB could be a valuable and safe option for the management of AWS in AUD patients affected by liver cirrhosis, also when administered early and even at higher than recommended dosages.

**Supplementary Information:**

The online version contains supplementary material available at 10.1007/s11739-024-03761-x.

## Introduction

Alcohol use disorders (AUD) are the most prevalent of all substance use disorders worldwide. The single-year prevalence globally has been estimated to be over 100 million individuals. Additionally, nearly 3 million deaths (5.3% of all deaths globally) have been attributed to alcohol-related mortality in a single year [1].

Alcohol withdrawal syndrome (AWS) is a distressing and life-threatening condition that affects AUD patients when they decrease or discontinue their alcohol consumption. The most common symptoms include tremor, paroxysmal sweats, nausea, vomiting, insomnia, tachycardia, hallucinations, agitation, and restlessness. In severe cases, symptoms might progress to seizures, *delirium tremens* and coma or even cardiac arrest and death in 5% to 10% of people [2].

The main aim of AWS treatment is to minimize the severity of symptoms in order to prevent the onset of complications. The tool most commonly used to assess AWS severity is the Clinical Institute of Withdrawal Assessment for Alcohol Scale (CIWA-Ar) [3]. Benzodiazepines (BZDs) are considered the “gold standard” therapy for AWS treatment and, among them, diazepam is the first choice. However, particular care should be taken in frail subjects, i.e., patients affected by liver impairment since BZDs with long half-life can accumulate and cause serious adverse events (AEs). In these cases, BZDs with a shorter half-life are to be preferred [4–6]. As an alternative to BZDs, other compounds such as drugs acting on the gamma-aminobutyric acid (GABA) system (i.e., sodium oxybate, baclofen, chlormethiazole, and anticonvulsants), and dopamine antagonists (i.e., tiapride) have been studied for AWS treatment [6]. Sodium oxybate, also called gamma-hydroxybutyric acid (GHB), is a short-chain fatty acid physiologically present in the central nervous system. GHB is structurally similar to the GABA neurotransmitter and binds to the GABA-B receptor. It has alcohol-mimicking effects, and it has been tested in pre-clinical and clinical settings for AWS treatment with satisfactory results. GHB is largely absorbed after oral administration; it acts rapidly, reaching a plasma concentration peak in 30–120 min, and its half-life is around 30–60 min. It is mainly metabolized by the liver and only a small part (around 2–5%) is excreted unchanged in the urine [6]. The main pharmacological indication of GHB is for narcolepsy treatment and it has been approved for AWS treatment in Austria and Italy only. According to the Italian Society of Alcoholism (SIA) guidelines, the daily GHB dosage for AWS treatment has to be 50–100 mg/kg divided into 3–6 daily administrations [7].

Liver cirrhosis is defined as the histological development of regenerative nodules surrounded by fibrous bands in response to chronic liver injury, which leads to portal hypertension and end-stage liver disease. The major clinical consequences of cirrhosis are impaired hepatocyte function, an increased intrahepatic resistance and the development of hepatocellular carcinoma. Alcoholic liver disease and hepatitis C are the most common causes in the western hemisphere region [8].

The hepatic impairment caused by liver cirrhosis reduces the organ’s ability to metabolize many xenobiotics (AWS therapy included), leading to the possible onset of clinically relevant AEs. In this context, the present study aimed to evaluate and describe the use of GHB for AWS in hospitalized patients with diagnosis of liver cirrhosis.

## Methods

### Study design and setting

An observational retrospective study on 166 patients affected by liver cirrhosis and AUD was performed. Patients were hospitalized for AWS in the Medical Toxicology Unit of Careggi University Hospital in Florence (Italy), from January 1, 2005 to December 31, 2015. The cohort of patients suitable for enrollment was retrieved from the hospital’s electronic database. The protocol of the study was approved by the local Ethic Committee (Comitato Etico Regione Toscana—Area Vasta Centro, CEAVC (Italy); Prot. n. 0001510/2024, 21,680/oss; January 22, 2024).

Inclusion criteria were: (1) hospitalization for AWS and (2) diagnosis of liver cirrhosis. Exclusion criteria were: (1) liver cirrhosis diagnosis not confirmed; (2) onset of *delirium tremens*; (3) patients transferred from/to other hospitals or units without the possibility to obtain the necessary information regarding pharmacological treatment.

Demographical (sex and age) and clinical characteristics (hematologic parameters for liver function, presence of hepatitis virus C and B, other substance use disorders) were retrieved for all patients included in the cohort. Hepatitis screening was conducted upon admission to the hospital. Alternatively, if the hepatitis test had been performed before admission, positivity for hepatitis was recorded in the patient’s medical history.

### Alcohol withdrawal syndrome assessment

The presence and the severity of AWS were assessed using the CIWA-Ar scale [3]. The CIWA-Ar scale examines 10 clinical parameters: nausea and vomiting, agitation, anxiety, hearing disorders, visual disorders, sensory alterations, headache, sweating, tactile disorders, and tremors. All parameters used for CIWA-Ar scale calculation were reported in the patients’ clinical charts. This allowed for the CIWA-Ar calculation to be performed even after hospital admission. The score obtained by the assessment of all parameters allows us to identify 4 degrees of AWS severity: grade 1 (≤ 9) very mild withdrawal; grade 2 (10–15) mild AWS; grade 3 (16–20) modest AWS, grade 4 (≥ 21) severe withdrawal. When not clearly reported in medical records, CIWA-Ar score was calculated by a single medical toxicologist in order to minimize interpretative bias. CIWA-Ar score was assessed both at the patient’s admission (CIWA-Ar T_0_) and at the time of AWS maximum severity (CIWA-Ar _Max_). For the present analyses, patients were divided into two groups based on their different CIWA-Ar scores: patients with CIWA-Ar 1 and 2 (very mild/mild), and patients with CIWA-Ar 3 and 4 (modest/severe).

### Liver cirrhosis assessment

Patients underwent blood tests and instrumental tests before or during hospitalization. The diagnosis of liver cirrhosis was achieved using clinical records. When liver biopsy was not clearly reported, the diagnosis had to be confirmed by a single hepatologist who valued the presence of the following data (criteria) retrieved both in previous clinical history and from in-hospital assessment: liver stiffness measurement (Fibroscan), ultrasound criteria, such as hypertrophy of the left lobe, irregular or lumpy margins, dilation of the portal trunk, splenomegaly, and collateral circulation when echography was performed, laboratory (biohumoral) alterations, such as thrombocytopenia hypoalbuminemia, increased INR and jaundice as signs of liver failure, skin alterations, such as spider naevi and palmar erythema, ascites, esophageal varices (with or without bleeding), and hepatic encephalopathy. A single hepatologist evaluated all clinical records to confirm the diagnosis in order to minimize interpretative bias. Moreover, the inclusion and exclusion criteria, the careful evaluation of clinical parameters and the assessment performed by the hepatologist ensured the accurate distinction between cirrhosis and acute alcoholic hepatitis, allowing the exclusion of patients affected by alcoholic hepatitis from our study. The severity of liver cirrhosis was assessed using three different standardized tools: CTP (Child–Turcotte–Pugh), MELD (Model for End-Stage Liver Disease) and MELD-Na (MELD-sodium) [9].

### Pharmacological treatments

Drugs used in our population to manage AWS included different kinds of BZDs, phenobarbital and GHB. All BZDs molecules and phenobarbital were converted to diazepam equivalents according to international standardized conversion tables [5]. We identified five therapeutic schemes based on the different kind of molecules used to treat AWS: (1) BDZ only; (2) BDZ and GHB; (3) BDZ and phenobarbital; (4) BDZ, phenobarbital and GHB; (5) phenobarbital and GHB. Furthermore, patients were divided in two groups based on GHB intake. Patients belonging to the first group were treated with GHB in association with other drugs, the second group was treated without GHB.

The occurrence of adverse events (AEs) possibly related to AWS pharmacological therapies was also retrieved. In particular, we focused on the occurrence of “drowsiness” as an AE, which was identified and coded using the Medical Dictionary for Regulatory Activities Terminology (MedDRA) classification system (code 10,013,649) [10].

### Statistical analysis

The compilation, archiving of electronic folders and data extrapolation was carried out using the “ARCHIMED” electronic registry (version 1.0) [11]. Categorical variables were described as numbers and percentages and compared with the chi-squared test, while continuous variables were presented as mean and standard deviation (SD) or median and interquartile range (IQR) and compared through the Student’s t or Kruskal–Wallis tests, respectively. The paired *t* test was used to compare the MELD and MELD-Na scores at admission and discharge for each subject in the database.

A multivariate logistic regression was performed to estimate the probability of having a CIWA-Ar Max 3–4 during hospitalization, an AWS longer than 36 h, a hospitalization longer than 9 days, and the probability of developing drowsiness as an AE during AWS treatment. These estimates were calculated between patients treated with GHB and those without GHB (reference group). Furthermore, an adjunctive multivariate logistic regression analysis was performed only for patients treated with GHB to evaluate the effect of its early administration (within the first day of hospitalization) on the abovementioned outcomes, and to estimate the probability of developing drowsiness with a GHB dose  ≥ 100 mg/kg and in presence of other central nervous system sedative agents (i.e., antipsychotics, methadone, and/or opioids of abuse). The odds ratios (ORs) were adjusted for age, sex, hepatitis virus positivity, presence of other substance use disorders, MELD score at admission, and blood alcohol concentration (BAC). Statistical significance was considered with a *p* value  ≤ 0.05. Statistical analyses were carried out using Stata 17 (StataCorp).

## Results

**Fig. 1:**
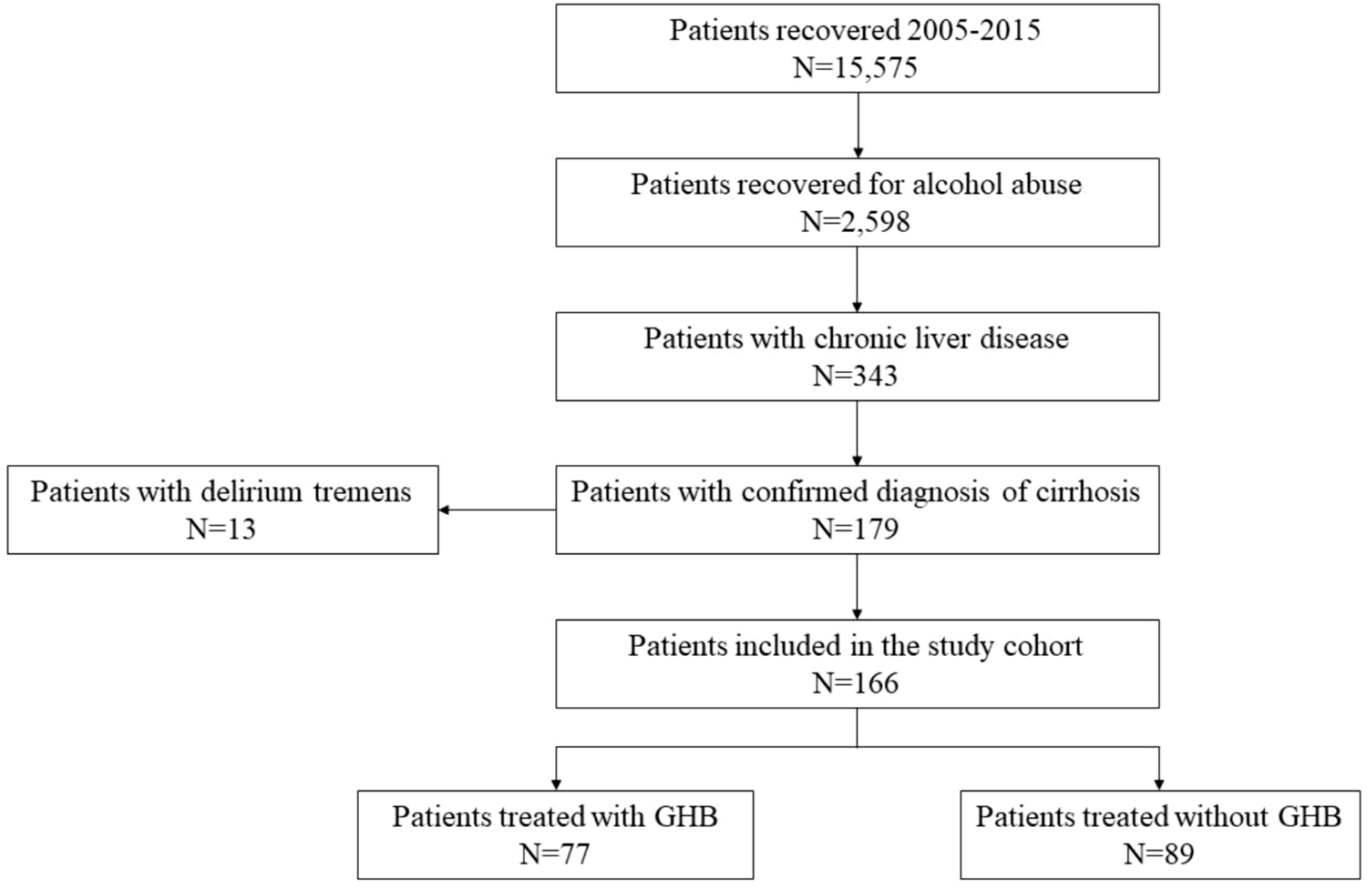
Inclusion of patients in the study cohort

From January 1, 2005 to December 31, 2015, 15,575 patients were admitted to the Medical Toxicology Department of Careggi University Hospital, Florence (Italy), 2598 of whom were hospitalized for AWS. Of these patients, 179 were suffering from liver cirrhosis. Thirteen patients were excluded due to the occurrence of *delirium tremens*. A total of 166 AUD patients were included in the present analysis, of these 77 subjects received GHB and 89 were treated without GHB (Fig. 1). Among those treated with GHB, the majority of them (*n* = 54, 70.13%) received the GHB-based therapy early after hospital admission, within the first day (*data not shown*).

Overall, the majority of AUD patients were ≥ 40 years of age (87.35%) and were males (80.12%) (Table 1). The two groups were homogeneous in terms of positivity to HBV or HCV, use of other substances of abuse, and the severity of liver cirrhosis assessed with Child–Pugh score (*p* > 0.05). On the contrary, parameters like the severity of liver cirrhosis assessed with MELD original score and MELD-Na score at patients’ admission and discharge, the mean alcohol units that patients declared to drink, the blood alcohol concentration (BAC) levels at patients’ arrival, the length of AWS and hospitalization, as well as the severity of AWS assessed by CIWA-Ar (CIWA-Ar_T0_, CIWA-Ar _Max_) score, resulted to be significantly higher among AUD patients treated with GHB (*p* < 0.05). This highlights that AUD patients treated with GHB presented a more complex clinical picture, both in terms of liver function and of alcohol abuse and relative withdrawal syndrome. All AUD patients treated with AWS treatments (with or without GHB) went through a complete resolution of withdrawal symptoms. Overall, MELD and MELD-Na scores significantly improved during hospital stay (15.76 ± 4.42 vs 14.06 ± 4.87 and 17.5 ± 5.17 vs 15.72 ± 5.86, respectively). In particular, MELD score significantly improved in both groups.

**Table 1:** Demographic and clinical characteristics of patients diagnosed with cirrhosis

	Overall *N* = 166 (%)	Patients treated with GHB *N* = 77 (%)	Patients treated without GHB *N* = 89 (%)	*p *value
Age (years)				
< 40	21 (12.65)	11 (14.29)	10 (11.24)	0.556
≥ 40	145 (87.35)	66 (85.71)	79 (88.76)	
Mean ± SD	47.28 ± 7.53	47.64 ± 7.76	46.98 ± 7.35	0.575
Median (IQR)	47 (43–50)	47 (44–50)	47 (43–50)	0.476
Sex				
Males	133 (80.12)	61 (79.22)	72 (80.90)	0.787
Females	33 (19.88)	16 (20.78)	17 (19.10)	
Positivity for hepatitis				
No	61 (36.75)	29 (37.66)	32 (35.96)	0.312
HCV	77 (46.39)	31 (40.26)	46 (51.69)	
HBV	11 (6.63)	7 (9.09)	4 (4.49)	
HCV and HBV	17 (10.24)	10 (12.99)	7 (7.87)	
Use of other substances of abuse^a^				
Yes	81 (48.80)	44 (57.14)	37 (41.57)	0.124
No	57 (34.34)	23 (29.87)	34 (38.20)	
Prior to admission	28 (16.87)	10 (12.99)	18 (20.22)	
Concomitant use of other CNS sedatives				
No	67 (40.36)	38 (49.35)	29 (32.58)	0.028
Yes	99 (59.64)	39 (50.65)	60 (67.42)	
Methadone	27	11	16	
Antipsychotics	41	15	26	
Methadone and antipsychotics	30	12	18	
Opioids	1	1	–	
CTP score				
A	60 (36.14)	24 (31.17)	36 (40.45)	0.463
B	80 (48.19)	40 (51.95)	40 (44.94)	
C	26 (15.66)	13 (16.88)	13 (14.61)	
Mean ± SD	7.51 ± 2.08	7.71 ± 2.09	7.35 ± 2.07	0.280
Median (IQR)	7 (6–9)	8 (6–9)	7 (6–9)	0.221
MELD scores				
MELD at admission				
Mean ± SD	12.69 ± 5.01	13.67 ± 5.74	11.72 ± 3.97	0.017
Median (IQR)	12 (9–15)	13 (10–17)	11.5 (8.5–15)	0.029
MELD-Na at admission				
Mean ± SD	14.67 ± 5.33	15.85 ± 6.13	13.48 ± 4.10	0.006
Median (IQR)	14 (11–18)	15 (12–20)	13 (10–16)	0.012
MELD at discharge				
Mean ± SD	13.89 ± 5.00	15.06 ± 5.88	12.95 ± 4.05	0.200
Median (IQR)	14 (10–17)	15 (11–18)	12 (10–15)	0.257
MELD-Na at discharge				
Mean ± SD	15.82 ± 5.87	16.56 ± 7.15	15.19 ± 4.60	0.477
Median (IQR)	17 (10–19)	18 (10–21)	16 (12–19)	0.404
MELD at admission vs MELD at discharge^b^				
MELD at admission	15.76 ± 4.42	16.65 ± 4.43	14.88 ± 4.36	–
MELD at discharge	14.06 ± 4.87	15.06 ± 5.88	13.06 ± 3.49	–
* p-*value	< 0.001	0.019	0.018	–
MELD-Na at admission vs MELD-Na at discharge^b^				
MELD-Na at admission	17.5 ± 5.17	18.89 ± 5.31	16.11 ± 4.78	–
MELD-Na at discharge	15.72 ± 5.86	16.56 ± 7.16	14.89 ± 4.25	–
* p-*value	0.026	0.087	0.172	–
Declared alcohol consumption (IU/die)				
Mean ± SD	19.96 ± 12.25	22.34 ± 14.57	17.94 ± 9.52	0.039
Median (IQR)	16 (12–26.7)	20 (13.35–29.2)	16 (10–26)	0.129
BAC at admission (g/L)				
Mean ± SD	1.76 ± 1.39	2.42 ± 1.36	1.36 ± 1.26	< 0.001
Median (IQR)	1.7 (0.51–2.64)	2.36 (1.34–3.07)	1.23 (0–2.43)	< 0.001
AWS duration (days)				
Mean ± SD	1.54 ± 0.74	1.82 ± 0.81	1.29 ± 0.57	< 0.001
Median (IQR)	1 (1–2)	2 (1–2)	1 (1–1)	< 0.001
Hospitalization length (days)				
Mean ± SD	9.14 ± 4.27	10.17 ± 4.09	8.25 ± 4.25	0.003
Median (IQR)	8 (6–10)	9 (8–12)	7 (6–9)	< 0.001
AWS severity				
CIWA-Ar _T0_				
Mean ± SD	3.20 ± 5.28	4.73 ± 6.30	1.89 ± 3.77	< 0.001
Median (IQR)	0 (0–6)	0 (0–9)	0 (0–0)	0.002
CIWA-Ar _Max_				
Mean ± SD	10.46 ± 4.92	13.57 ± 4.34	7.76 ± 3.64	< 0.001
Median (IQR)	10 (6–14)	13 (10–16)	6 (6–9)	< 0.001

*BAC* blood alcohol concentration, *CTP* Child–Turcotte–Pugh, *GHB* gamma-hydroxybutyric acid, *HBV* hepatitis B virus, *HCV* hepatitis C virus, *IQR* interquartile range, *MELD* model for end-stage liver disease, *SD* standard deviation

^a^Referred to opioids, cannabis and cocaine

^b^Paired *t* test comparison (mean ± SD)

Considering AUD patients treated with GHB (Table 2), its average daily dose amounted to 53.04 mL, corresponding to about 88.43 mg/kg. During home treatment (after hospital discharge), the dose was reduced to about 36.67 mL. In this group of patients, the reduction of GHB dose generally started from the 3rd day of therapy, which had an average duration of 6 days. About 15.6% of AUD patients treated with GHB discontinued therapy due to drowsiness.

**Table 2:** Characteristics of GHB treatment

	Patients treated with GHB *N* = 77 (%)
Daily dose of GHB (mL)	
Mean ± SD	53.04 ± 33.96
Median (IQR)	40 (30–70)
Daily dose of GHB on body weight (mg/kg)	
Mean ± SD	88.43 ± 34.84
Median (IQR)	81.5 (64–106.5)
Daily dose of GHB at home (mL)	
Mean ± SD	36.67 ± 5.00
Median (IQR)	40 (30–40)
Start of GHB dose reduction (starting on the day)	
Mean ± SD	3.21 ± 0.94
Median (IQR)	3 (3–4)
Duration of GHB therapy (days)	
Mean ± SD	6.16 ± 2.00
Median (IQR)	6 (5–7)
Withdrawal of GHB due to drowsiness	
Yes	12 (15.58)
No	64 (83.12)
Missing	1 (1.30)

*GHB* gamma-hydroxybutyric acid, *IQR* interquartile range, *SD* standard deviation

Observing the severity of AWS (Table 3), patients with CIWA-Ar scores 3–4 (severe) received a significantly higher mean daily dose of GHB both at the time of hospital admission (87.75 vs 51.14 mL) and during the hospitalization (68.70 vs 47.54 mL). Furthermore, these patients also showed a significantly longer duration of AWS both at the time of hospital admission (3 vs 1.75 days) and during the hospitalization (2.35 vs 1.63 days).

**Table 3:** CIWA-Ar, duration of hospitalization and of AWS in patients treated with GHB

	CIWA-Ar _T0_		*p-*value	CIWA-Ar _Max_		*p *value
	CIWA-Ar 1–2 *N* = 73	CIWA-Ar 3–4 *N* = 4		CIWA-Ar 1–2 *N* = 57	CIWA-Ar 3–4 *N* = 20	
Daily dose of GHB (mL)						
Mean ± SD	51.14 ± 29.91	87.75 ± 77.76	0.035	47.54 ± 26.75	68.70 ± 46.42	0.015
Median (IQR)	40 (30–70)	65 (40.5–135)	0.355	40 (30–60)	55 (40–85)	0.031
Daily dose of GHB on body weight (mg/kg)						
Mean ± SD	88.13 ± 34.43	96.5 ± 61.52	0.742	89.62 ± 36.64	83.54 ± 27.13	0.609
Median (IQR)	81.5 (65–106)	96.5 (53–140)	0.877	82 (63–107)	78 (67–95)	0.549
Daily dose of GHB at home (mL)						
Mean ± SD	36.25 ± 5.17	–	–	36.67 ± 5.16	36.67 ± 5.77	1.00
Median (IQR)	40 (30–40)	–	–	40 (30–40)	40 (30–40)	1.00
Start of GHB dose reduction (starting on the day)						
Mean ± SD	3.22 ± 0.95	3.0 ± 0.82	0.652	3.10 ± 0.94	3.5 ± 0.89	0.105
Median (IQR)	3 (3–4)	3 (2.5–3.5)	0.694	3 (3–4)	3 (3–4)	0.086
Duration of GHB therapy (days)						
Mean ± SD	6.17 ± 2.05	6.00 ± 0.82	0.872	6.02 ± 1.89	6.55 ± 2.82	0.310
Median (IQR)	6 (5–7)	6 (5.5–6.5)	0.981	6 (5–7)	6 (5–7.5)	0.472
Duration of hospitalization (days)						
Mean ± SD	10.09 ± 4.03	11.50 ± 5.69	0.508	10.14 ± 4.22	10.25 ± 3.82	0.919
Median (IQR)	9 (8–12)	9 (8.5–14.5)	0.747	9 (7–12)	9 (8–13.5)	0.829
Duration of AWS (days)						
Mean ± SD	1.75 ± 0.76	3.00 ± 0.82	0.002	1.63 ± 0.70	2.35 ± 0.87	< 0.001
Median (IQR)	2 (1–2)	3 (2.5–3.5)	0.009	2 (1–2)	2 (2–3)	0.001

*AWS* alcohol withdrawal syndrome, *GHB* gamma-hydroxybutyric acid, *IQR* interquartile range, *SD* standard deviation

Other drugs were also administered to manage AWS (Table 4). In particular, AUD patients with cirrhosis treated with GHB received a significantly lower mean dose of concomitant lorazepam (0.66 vs 3.42 mg).

**Table 4:** Concomitant medications used to treat AWS

	Overall *N* = 166 (%)	Patients treated with GHB *N* = 77 (%)	Patients treated without GHB *N* = 89 (%)	*p *value
Concomitant medications				
BZD	71 (42.77)	14 (18.18)	57 (64.04)	–
Phenobarbital	6 (3.61)	6 (7.79)	–	
BZD and phenobarbital	89 (53.62)	57 (74.03)	32 (35.96)	
Dosage of concomitant medications				
Chlordiazepoxide (mg)				
Mean ± SD	90.21 ± 58.15	104.07 ± 68.00	81.61 ± 49.60	0.252
Median (IQR)	80 (60–120)	90 (60–150)	60 (60–90)	0.063
Lorazepam (mg)				
Mean ± SD	1.51 ± 2.06	0.66 ± 1.61	3.42 ± 1.67	< 0.001
Median (IQR)	0 (0–2.5)	0 (0–0)	2.5 (2.5–5)	< 0.001
Diazepam (mgEq)				
Mean ± SD	62.99 ± 50.94	73.83 ± 60.65	54.34 ± 39.91	0.016
Median (IQR)	48 (24–86)	54 (22–110)	42 (24–73)	0.141
Diazepam (mgEq/die)				
Mean ± SD	39.88 ± 24.73	38.68 ± 29.21	40.84 ± 20.60	0.584
Median (IQR)	36.35 (23.25–53.35)	32 (16–52.7)	37 (24–54)	0.135
Phenobarbital (mgEq/die)				
Mean ± SD	96.67 ± 40.47	95.24 ± 33.99	99.48 ± 51.41	0.632
Median (IQR)	100 (50–100)	100 (100–100)	100 (50–100)	0.729

*AWS* alcohol withdrawal syndrome, *BZD* benzodiazepines, *GHB* gamma-hydroxybutyric acid, *IQR* interquartile range, *SD* standard deviation

The multivariate logistic regression (Table 5) showed that AUD patients treated with GHB were more likely to have a CIWA-Ar _Max_ 3–4 during hospitalization (OR 3.76 [CI 95% 1.02–13.85]), and a longer hospitalization (OR 3.08 [95% CI 1.23–7.71]). An AWS longer than 36 h (OR 2.32 [95% CI 0.92–5.85]) was also observed for this subset, although not to a statistically significant level. Moreover, compared to AUD patients who were not treated with GHB, those treated with GHB were less likely to develop drowsiness during AWS treatment (OR 0.74 [95% CI 0.23–2.38]). The level of significance was not reached for this estimate as well.

**Table 5:** Association of GHB treatment with severity and duration of AWS, length of hospitalization, and occurrence of drowsiness

	Crude OR (CI 95%)	Adjusted OR (CI 95%)
CIWA-Ar _Max_ 3–4 (*n* = 26)		
GHB vs no GHB	6.12 (2.18–17.16)	3.76 (1.02–13.85)
Early GHB vs late GHB	0.29 (0.10–0.86)	0.06 (0.01–0.49)
AWS > 36 h (*n* = 67)		
GHB vs no GHB	4.59 (2.35–8.93)	2.32 (0.92–5.85)
Early GHB vs late GHB	0.21 (0.06–0.70)	0.20 (0.04–1.09)
Hospitalization > 9 days (*n* = 53)		
GHB vs no GHB	3.58 (1.79–7.15)	3.08 (1.23–7.71)
Early GHB vs late GHB	0.44 (0.16–1.20)	1.45 (0.33–6.30)
Occurrence of drowsiness (*n* = 31)		
GHB vs no GHB	0.66 (0.30–1.46)	0.74 (0.23–2.38)
Early GHB vs late GHB	0.54 (0.15–1.91)	0.48 (0.04–5.24)
GHB dose ≥ 100 mg/kg/die vs < 100 mg/kg/die	0.71 (0.21–2.48)	1.50 (0.21–10.89)
Presence of other CNS sedatives^a^ vs no other sedatives	7.22 (1.46–35.61)	–

The analyses were adjusted for age, sex, hepatitis virus positivity, presence of other substance use disorders, MELD score at admission, and blood alcohol concentration (BAC). The number of patients achieving each outcome is reported in brackets

*AWS* alcohol withdrawal syndrome, *CI* confidence interval, *CNS* central nervous system, *GHB* gamma-hydroxybutyric acid, *OR* odds ratio

^a^Antipsychotics, methadone, and/or opioids

An adjunctive logistic regression analysis showed that, among AUD patients treated with GHB, the early administration of GHB (within the first day of hospitalization) significantly decreased the probability of CIWA-Ar _Max_ worsening during the hospitalization (OR 0.06 [95% CI 0.01–0.49]). Furthermore, the probability of developing drowsiness with a dose of GHB  ≥ 100 mg/kg was not significantly higher than at lower doses of this drug, while AUD patients exposed to other central nervous system sedative agents were more likely experiencing drowsiness (OR 7.22 [95% CI 1.46–35.61]).

Supplementary Table 1 shows the main clinical features of cirrhosis. Most AUD patients with cirrhosis have 2 to 4 complications (72.3%). Splenomegaly was reported in approximately 60% of patients and thrombocytopenia in 82% of them. Among the clinical variables considered, a statistically significant difference was observed for thrombocytopenia (70 vs 67 cases) and for bleeding from esophageal varices (8 vs 6 cases), that were more frequent in patients treated with GHB (*p* < 0.05).

Details of the blood tests carried out during hospitalization by AUD patients with cirrhosis are shown in Supplementary Table 2. Among the clinical variables considered, a statistically significant difference was observed for the gamma-glutamyltransferase (425.43 vs 638.41 UI/L) and the international normalized ratio (1.31 vs 1.40) levels, that resulted to be lower in the GHB subgroup (*p* < 0.05).

## Discussion

The present retrospective observational study described the use of GHB for the treatment of AWS in hospitalized AUD patients diagnosed with liver cirrhosis, thus representing valuable new insights into the pharmacological management of AWS in a clinically frail subgroup.

Long-acting BZDs are known to be the gold standard therapy for AWS [12]. Drugs with shorter half-life, such as short-acting BZDs or GHB, should be chosen in case of hepatic impairment to avoid drug accumulation and potentially life-threatening AEs [13–15]. The utility of GHB as a treatment for AWS is largely demonstrated by many studies [7, 16–18, 18–22], nevertheless none of them focused the attention on the use of this drug in AUD patients affected by liver cirrhosis. In fact, in the scientific literature, only one case report is described evaluating the use of GHB in a patient affected by liver cirrhosis and concurrent AWS [23]. In their anecdotal description, Caputo et al. concluded that physicians should always consider GHB as a valuable non-BZD GABA-ergic pharmacological option for the treatment of AWS even in AUD patients with a severe liver disease, especially those with alcohol dependence who experienced AWS in the hospital setting. On this regard, a study published in 1996 analyzed the pharmacokinetics changes of GHB in patients affected by moderate and severe hepatic impairment [24], confirming that liver failure can cause many alterations in GHB pharmacokinetics, such as half-life prolongation and clearance reduction, but none of those are sufficient to induce an accumulation of the drug when administered in repeated doses.

Considering the clinical setting of AWS, the efficacy and the safety of GHB were already evaluated in several Italian and Austrian studies. In particular, a single-blind trial comparing GHB versus diazepam did not show a significantly different efficacy of these drugs in suppressing AWS [17], even though GHB reduced anxiety, agitation and current depression more rapidly than diazepam. Other investigations, however, demonstrated that GHB was even more effective than diazepam in treating AWS [25], and equally efficient as clomethiazole [26]. GHB efficacy was further confirmed by treating AWS in almost 300 hospitalized AUD patients affected by different clinical conditions [27]. Nevertheless, these conditions did not include liver impairment. Compared to our sample, in all these studies, GHB was administered at the dose of 50–100 mg/kg divided into three or four daily administrations, and no safety issues were observed. In 2010, a Cochrane systematic review was performed to evaluate the efficacy and safety of GHB for treatment of AWS [7]. Authors included randomized controlled trials and controlled prospective studies evaluating GHB vs placebo or other pharmacological treatments (including diazepam). Of note, there was insufficient randomized evidence to be confident that the effects of GHB and placebo were different, or to determine reliably if GHB was more or less effective than other drugs for the treatment of AWS. In fact, comparing GHB 50 mg with diazepam, the CIWA‐Ar scores for tremor, anxiety and agitation were more often lower for GHB, but the difference was usually not statistically significant. No significant differences were found between the drugs for AEs. To date, despite this cluster of evidence, studies are still limited and investigations including a larger number of patients are needed. In addition, some safety concerns, such as potential development of GHB dependence, have to be more investigated [28]. According to recent literature reviews concerning the management of AUD in patients with alcohol-associated liver disease, GHB may be considered a treatment option when other drugs are deemed as not appropriate but, as confirmed by our evidence, results regarding its use in clinical practice are promising [29, 30]. Additionally, its usefulness has also been demonstrated in maintaining abstinence. In fact, abstinence rates have increased by up to 34% and pharmacovigilance analyses have reported very few adverse side effects and only a few cases of abuse [31].

Based on our clinical practice routine, the use of GHB is influenced by AWS severity, alcohol tolerance and the presence, concomitant or previous, of other substances of abuse, in particular opioids. Similarly, AUD patients diagnosed with liver cirrhosis, affected by more severe AWS and with a higher alcohol tolerance, are more frequently treated with GHB. In particular, we observed that AUD patients who presented higher values of liver impairment were more likely to be treated with GHB. Regarding the use of GHB in patients known to be poly-abusers, concern has been raised regarding the risk of developing GHB addiction, misuse or abuse [32]. Thus, it is important to underline that GHB must always be used in a controlled environment (hospital), under the strict supervision of specialist doctors (clinical toxicologists). In addition, continuous monitoring of clinical parameters, such as CIWA-Ar, MELD, and CPT scores, as well as the severity of AWS, plays a pivotal role in guiding the management of GHB therapy. Ensuring strict control and regular assessment of these parameters is essential for optimizing treatment outcomes and minimizing potential risks associated with GHB administration. In our sample, although with a slightly longer hospital stay, all AUD patients with cirrhosis treated with GHB went through a complete resolution of withdrawal symptoms. However, it is already described in the scientific literature that the early administration of GHB can prevent the worsening of the syndrome [7]. In light of these considerations, the timing of GHB administration can be considered a relevant key point for an appropriate management of AWS. In fact, among the patients managed at our Medical Toxicology Unit who were administered with GHB early during the first day of hospitalization, we observed a significantly lower probability of AWS worsening. The duration of AWS was slightly longer for AUD patients treated with GHB and it is probably due to the more severe syndrome at arrival that required a more intensive and prolonged treatment. Moreover, these patients also had a longer period of stay (> 36 h) in our Medical Toxicology Unit, and this could be related to the necessity to gradually taper the dosage of GHB until complete discontinuation before discharge. From our analysis, in a small percentage of AUD patients, GHB is associated with lower efficacy when it is not administered promptly on the first day of hospitalization. However, in abuser or poly-abuser patients in the presence of pathological cirrhosis, GHB still guarantees good results both in terms of manageability and safety for patients.

Excessive sedation (i.e., drowsiness) is the only AE related to drug administration that occurred in our sample. The onset of this AE required the reduction or the discontinuation of one or more drugs to achieve the complete resolution of the symptom. We tried to identify the risk factors for the onset of excessive sedation. Analyzing our data, we found out that GHB therapy was not related to a higher risk of drowsiness. The data analysis confirmed that the dosage of GHB (mg/Kg/die) was not related to the onset of excessive sedation, not even when the dosage used was higher than the recommended one (50–100 mg/Kg/die). On the contrary, it appeared that the administration of drugs acting on the CNS, such as neuroleptics, methadone, and opioids, was related to a higher risk of excessive sedation. This safety evidence confirms that, if administered in a hospital setting, GHB can be considered a safe therapeutic option, also for frail AUD patients, such as those affected by liver impairments.

We also tried to assess if the administration of withdrawal therapy had any negative impact on liver function. All patients went through an improvement of both scores. This result shows that AWS treatments did not cause liver cirrhosis worsening in both groups. The improvement of MELD values was probably due to the complete alcohol abstention during the hospital stay.

### Limitations and strengths

This study has several limitations. The main one is represented by the small sample size and the retrospective nature of the study design. Both these aspects may have led to a poor characterization and an underestimation of GHB usefulness for AWS treatment, both in terms of effectiveness and safety. Moreover, the concomitant use of GHB with other drugs, including benzodiazepines and phenobarbital, does not allow us to draw firm conclusions on GHB efficacy. Furthermore, it was not possible to address if, compared to patients treated with standard therapy (i.e., BZDs), those administered with GHB had a higher decrease of cirrhosis severity scores. Finally, the limited follow-up did not allow to asses if GHB-treated AUD patients were more likely to experience a relapse compared to those treated with standard therapy.

On the other hand, this is the first real-world analysis performed in AUD patients affected by cirrhosis, describing the effect of time and dose of administration of GHB in AWS on several relevant outcomes that may support clinicians in their clinical practice.

## Conclusions

The present real-world analysis underlines that GHB could be a valuable and safe option for the management of AWS in AUD patients affected by liver cirrhosis, especially when administered early and even at higher than recommended dosages. This approach is considered innovative also by the recent international guidelines for the management of patients affected by alcohol-associated liver disease [33]. However, the evidence for its efficacy is still weak, mainly due to the low sample size of the studies carried out. To date, it seems a priority to carry out good quality randomized trials, with an adequate sample size, evaluating standardized outcome measures, homogeneous evaluation scales and common times in drug administration.

## Supplementary Information

Below is the link to the electronic supplementary material.

Supplementary file1 (DOCX 30 KB)

## Data Availability

The datasets generated during and/or analysed during the current study are available from the corresponding author on reasonable request.
