# Supplementary material for: Real-world analysis on the use of gamma-hydroxybutyric acid for alcohol withdrawal syndrome in hospitalized patients with diagnosis of cirrhosis
Source: Intern Emerg Med. 2024 Sep 9;20(1):119–29. doi: 10.1007/s11739-024-03761-x (PMC11794374; doi:10.1007/s11739-024-03761-x)
Supplement: Supplementary file 1 — Supplementary file1 (DOCX 30 KB) [file 11739_2024_3761_MOESM1_ESM.docx]

**Supplementary Table 1 -** Clinical features of cirrhosis.

|  | **Overall**  **N=166 (%)** | **Patients treated**  **with GHB**  **N=77 (%)** | **Patients treated**  **without GHB**  **N=89 (%)** | ***p-value*** |
| --- | --- | --- | --- | --- |
| **Number of complications** | | | | |
| None | 5 (3.01) | 1 (1.30) | 4 (4.49) | 0.168 |
| 1 | 20 (12.05) | 11 (14.29) | 9 (10.11) |  |
| 2-4 | 120 (72.29) | 59 (76.62) | 61 (68.54) |  |
| 5 or more | 21 (12.65) | 6 (7.79) | 15 (16.85) |  |
| **Dilatation of the splenoportal axis** | | | | |
| Yes | 52 (31.33) | 21 (27.27) | 31 (34.83) | 0.295 |
| No | 114 (68.67) | 56 (72.73) | 58 (65.17) |  |
| **Splenomegaly** | | | | |
| Yes | 99 (59.64) | 45 (58.44) | 54 (60.67) | 0.770 |
| No | 67 (40.36) | 32 (41.56) | 35 (39.33) |  |
| **Lumpy hepatic margins** | | | | |
| Yes | 58 (34.94) | 28 (36.36) | 30 (33.71) | 0.720 |
| No | 108 (65.06) | 49 (63.64) | 59 (66.29) |  |
| **Steatosis** | | | | |
| Yes | 58 (34.94) | 23 (29.87) | 35 (39.33) | 0.248 |
| No | 106 (63.85) | 52 (67.53) | 54 (60.67) |  |
| *Missing* | 2 (1.21) | 2 (2.60) | - |  |
| **Left lobe hypertrophy and hypotrophy of the right lobe** | | | | |
| Yes | 69 (41.57) | 35 (45.45) | 34 (38.20) | 0.344 |
| No | 97 (58.43) | 42 (54.55) | 55 (61.80) |  |
| **Thrombocytopenia** | | | | |
| Yes | 137 (82.53) | 70 (90.91) | 67 (75.28) | 0.008 |
| No | 29 (17.47) | 7 (9.09) | 22 (24.72) |  |
| **Oesophageal varices** | | | | |
| Yes | 64 (38.55) | 30 (38.96) | 34 (38.20) | 0.182 |
| No | 81 (48.79) | 47 (61.04) | 34 (38.20) |  |
| *Missing* | 21 (12.66) | - | 21 (23.60) |  |
| **Variceal bleeding** | | | | |
| Yes | 14 (8.43) | 8 (10.39) | 6 (6.74) | 0.001 |
| No | 150 (90.36) | 69 (89.61) | 81 (91.01) |  |
| *Missing* | *2 (1.21)* | - | *2 (2.25)* |  |
| **Ascites** | | | | |
| Yes | 49 (29.52) | 27 (35.06) | 22 (24.72) | 0.274 |
| *Moderate* | *36* | *19* | *17* |  |
| *Severe* | *13* | *8* | *5* |  |
| No | 116 (69.88) | 49 (63.64) | 67 (75.28) |  |
| *Missing* | 1 (0.60) | 1 (1.30) | - |  |
| **Encephalopathy** | | | | |
| Yes | 31 (18.67) | 20 (25.97) | 11 (12.36) | 0.069 |
| *1* | *23* | *16* | *7* |  |
| *2* | *7* | *4* | *3* |  |
| *3* | *1* | *-* | *1* |  |
| No | 135 (81.33) | 57 (74.03) | 78 (87.64) |  |

**Supplementary Table 2 -** Hematologic parameters.

|  | **Overall**  **N=166 (%)** | **Patients treated**  **with GHB**  **N=77 (%)** | **Patients treated**  **without GHB**  **N=89 (%)** | ***p-value*** |
| --- | --- | --- | --- | --- |
| **AST (UI/L)** |  |  |  |  |
| Mean±SD | 135.09±90.03 | 123.59±81.84 | 148.38±97.50 | 0.077 |
| Median (IQR) | 113.5 (83-161) | 108 (73-154) | 133 (94-180) | 0.076 |
| **ALT (UI/L)** |  |  |  |  |
| Mean±SD | 65.03±51.32 | 65.79±54.27 | 64.16±48.03 | 0. 839 |
| Median (IQR) | 49 (33-74) | 49 (34-74) | 47 (33-73) | 0.705 |
| **GGT (UI/L)** |  |  |  |  |
| Mean±SD | 523.53±611.01 | 425.43±402.01 | 638.41±775.85 | 0.025 |
| Median (IQR) | 356 (180-626) | 298 (173-475) | 434 (196.5-865) | 0.031 |
| **Albumin (mg/dL)** |  |  |  |  |
| Mean±SD | 3.21±0.60 | 3.23±0.55 | 3.19±0.65 | 0.658 |
| Median (IQR) | 3.21 (2.86-3.65) | 3.19 (2.9-3.62) | 3.24 (2.64-3.70) | 0.709 |
| **INR** |  |  |  |  |
| Mean±SD | 1.35±0.27 | 1.31±0.25 | 1.40±0.28 | 0.035 |
| Median (IQR) | 1.30 (1.1-1.5) | 1.10 (1.2-1.5) | 1.40 (1.20-1.60) | 0.029 |
| **Platelets (U/µl)** |  |  |  |  |
| Mean±SD | 93.94±59.34 | 98.26±55.51 | 88.95±63.49 | 0.315 |
| Median (IQR) | 77.5 (55-119) | 88 (53-132) | 74 (56-101) | 0.170 |
| **MCV (fL)** |  |  |  |  |
| Mean±SD | 96.11±8.71 | 95.13±9.13 | 97.26±8.12 | 0.116 |
| Median (IQR) | 96.1 (90.8-103) | 95.2 (90.4-101) | 97.5 (93.3-103) | 0.084 |
| **Ammonium (µmol/l)** |  |  |  |  |
| Mean±SD | 124.52±54.76 | 124.82±51.57 | 124.13±59.18 | 0.945 |
| Median (IQR) | 114 (86-160) | 114 (86-164) | 116.5 (86-149) | 0.776 |
| **Hyperammonemia** |  |  |  |  |
| Yes | 31 (18.67) | 62 (69.66) | 62 (80.50) | 0.260 |
| No | 124 (74.70) | 19 (21.35) | 12 (15.60) |  |
| *Missing* | *11 (6.63)* | *8 (8.99)* | *3 (3.90)* |  |
| **Creatinine (mg/dL)** |  |  |  |  |
| Mean±SD | 0.96±4.23 | 0.63±0.22 | 1.35±6.20 | 0.275 |
| Median (IQR) | 0.60 (0.49-0.70) | 0.60 (0.50-0.70) | 0.56 (0.49-0.70) | 0.543 |
| **Sodium (mEq/L)** |  |  |  |  |
| Mean±SD | 137.82±4.40 | 138.16±2.91 | 137.44±5.66 | 0.298 |
| Median (IQR) | 138 (136-140) | 138 (137-140) | 137 (135-140) | 0.356 |

ALT: alanine aminotransferase; AST: aspartate aminotransferase; GGT: gamma glutamyltransferase; GHB: gamma-hydroxybutyric acid; INR: international normalized ratio; IQR: interquartile range; IU/L: international units/litre; MCV: mean corpuscular volume; SD: standard deviation.
